# Does Breastfeeding Help to Reduce the Risk of Childhood Overweight and Obesity? A Propensity Score Analysis of Data from the KiGGS Study

**DOI:** 10.1371/journal.pone.0122534

**Published:** 2015-03-26

**Authors:** Maike Miriam Grube, Elena von der Lippe, Martin Schlaud, Anna-Kristin Brettschneider

**Affiliations:** Department of Epidemiology and Health Monitoring, Robert Koch Institute, Berlin, Germany; Institute of Preventive Medicine, DENMARK

## Abstract

**Background:**

Current studies suggest that the beneficial effect of breastfeeding on overweight and obesity may have been largely overestimated. We examined the relationship between >4 months of full breastfeeding and overweight/obesity in children living in Germany.

**Methods:**

We analyzed retrospectively collected data on breastfeeding from children aged 3–17 years who participated in the German Health Interview and Examination Survey for Children and Adolescents (KiGGS baseline study) between 2003 and 2006 (n = 13163). To minimize confounding, we applied propensity score matching and multivariate logistic regression analyses to estimate the effect of breastfeeding on childhood overweight and obesity.

**Results:**

Adjusted analyses of the matched dataset (n = 8034) indicated that children who were breastfed for <4 months had a significant reduction in the odds of overweight (OR 0.81 [95% CI 0.71–0.92]) and obesity (OR 0.75 [95% CI 0.61–0.92]) compared to children who were not breastfed or who were breastfed for a shorter duration. Further analyses stratified by age group showed that the association was strongest in children aged 7–10 years (OR 0.67 [95% CI 0.53–0.84] for overweight and OR 0.56 [95% CI 0.39–0.81] for obesity), while no significant effect could be seen in other age groups.

**Discussion:**

Our findings support the hypothesis that breastfeeding does have a beneficial effect on childhood overweight and obesity, although the effect seems to be strongest in children of primary school age.

## Background

Breastfeeding has frequently been reported to protect infants from infectious diseases such as diarrhea, lower respiratory infections, and acute otitis media, because breast milk contains nutrients, enzymes, hormones, antibodies, and growth factors that assist in combatting infectious agents [1–3]. Studies suggest that breastfeeding not only offers short-term benefits, but might also bear positive health effects that emerge in later childhood and adolescence by reducing the risk of atopic diseases [2,4,5] and type 2 diabetes [6] and by improving cognitive development [6,7]. Breastfeeding has also been said to reduce the risk of later overweight and obesity in children [2,6,8]. The prevalence of childhood overweight and obesity has strongly increased in recent decades [9], although since 1999 there has been a stabilization and even modest decline in most European countries [10,11]. In Germany, a slight downward trend in childhood obesity has been observed since 2004 [12,13]. Overweight and obesity in childhood are associated with a wide range of co-morbidities [14] Obesity has been shown to track from childhood into adulthood [15,16] and adult obesity is strongly associated with increased cardiovascular morbidity and mortality [17]. Different causal pathways might explain the association between breastfeeding and childhood overweight and obesity. Breastfed infants may learn to recognize feelings of satiety easier, leading to a better self-regulation of energy intake later in life [18,19]. Breast milk hormones that affect food intake regulation and energy balance may have an impact on body fat deposition [20]. In addition, most infant formulas contain more protein than breast milk, leading to a greater insulin response and resulting in an increased development of adipose tissue [6,19]. It has also been suggested that the impact of breastfeeding on obesity might be explained by later food preferences with breastfed children adapting more easily to new food than formula-fed children. This may be because the taste of breast milk varies with the mother’s food choices whereas the taste of infant formula is largely consistent [18]. However, evidence for the long-term effects of breastfeeding is largely conflicting and results of previous research strongly vary depending on the study design employed. Randomized controlled trials on breastfeeding effects are rare, because owing to the well-established short-term benefits of breastfeeding, allocating subjects to breastfeeding and non-breastfeeding groups would raise well-founded ethical concern. We know of only one cluster-randomized trial that examined the effects of assignment to a breastfeeding promotion program. The intervention strongly increased both breastfeeding duration and exclusivity, but findings revealed no impact on the prevalence of obesity at the age of 6 [21] and 11 years [22]. Evidence for the benefits of breastfeeding predominantly relies on data from observational studies, which often fail to address the high degree of confounding concerning women’s breastfeeding decisions. A large meta-analysis [6] showed that larger studies that accurately controlled for potential confounders reported less protective effects than smaller studies with inadequate adjustment. Furthermore, the evidence for the protective effect of breastfeeding mainly derives from studies conducted in high-income countries, where breastfeeding is closely associated with higher socioeconomic status, which is a considerable source of confounding [6].

However, different methods to improve causal inference in observational studies on breastfeeding benefits have been suggested, including the comparison of cohorts from countries with different confounding structures, the comparison of discordant siblings, and the application of propensity score methods. A comparison of breastfeeding effects in England and Brazil demonstrated that breastfeeding is associated with reduced risk of obesity in the English, but not in the Brazilian sample [23]. Findings from five low- and middle-income countries, in which breastfeeding is less socially patterned, confirmed these results and showed no significant association between breastfeeding and obesity [24]. Several studies compared sibling pairs in which only one sibling was breastfed to analyze the impact of breastfeeding on obesity, using fixed-effects models to account for unobserved heterogeneity across different families [25–29]. In only one of these studies were breastfed children and adolescents found to have a significantly lower body mass index compared with their non-breastfed siblings [28]. Two current studies from the United States applied propensity score methods, taking into account how likely study participants are to have been breastfed considering their observed baseline characteristics. Both studies reported either no [30] or only small and inconsistent [31] effects of breastfeeding on obesity and body mass index, respectively. Our objective was to examine the impact of breastfeeding on overweight and obesity using data from a nationally representative survey of children and adolescents living in Germany. We examined the effects of full breastfeeding for at least 4 months on the risk of overweight and obesity in later childhood and adolescence. Striving to reduce the effects of confounding in the association between breastfeeding and overweight/obesity to the greatest possible extent, we employed propensity score matching and considered a rich set of covariates.

## Methods

### Study population

We analyzed data from the baseline survey of the German Health Interview and Examination Survey for Children and Adolescents (KiGGS), a cross-sectional study conducted between 2003 and 2006 which was based on a nationally representative sample and aimed at obtaining comprehensive data on the health of children and adolescents living in Germany. Detailed information on the study design has been published elsewhere [32]. The two-stage sampling procedure involved the selection of 167 study locations from strata formed according to federal state, community type, and population size. In a second step, an equal number of children per birth cohort within the sample points was randomly selected from local population registries and invited to participate in the study. The response rate was 66.6%, and 17640 children and adolescents aged 0–17 years participated in the study (8654 girls and 8986 boys). The survey involved questionnaires filled in by parents and questionnaires for children aged 11 years and older, physical examinations and tests, a wide range of blood and urine testing, and a computer-assisted personal interview performed by trained study physicians. Informed written consent was obtained from all parents and from adolescents aged 14 years and older. In the current analyses, we included children and adolescents aged 3–17 years, if information on whether they had been breastfed and on weight and height was available (n = 13163).

### Ethics statement

The study was approved by the Charité—Universitätsmedizin Berlin ethics committee, and by the Federal Commissioner for Data Protection.

### Definition of variables

#### Breastfeeding

Parents of the KiGGS study participants were asked whether their children had been breastfed at all and how long they had been fully breastfed. Full breastfeeding was defined as either exclusive breastfeeding or predominant breastfeeding with additional supply of water or other fluids like tea or fruit juices. A binary variable (fully breastfed ≥4 months yes/no) was constructed for the propensity score analyses. We chose to dichotomize breastfeeding at 4 months, first because recommendations on breastfeeding in Germany commonly vary between 4 and 6 months of exclusive breastfeeding [33,34] and second because by doing so we obtained a fairly equal number of subjects in the exposed and the unexposed group, allowing a matching ratio of 1:1.

#### Overweight and obesity

Body height (standing height without shoes) was measured to the nearest 0.1 cm using a Harpenden stadiometer (Holtain Ltd., Crymych, UK) and body weight was measured to the nearest 0.1 kg with a calibrated scale (Seca, Hamburg, Germany) with the child wearing only underwear. The body mass index (BMI) was calculated as the ratio of weight in kilograms by height in meters squared. Overweight was defined as BMI >90^th^ percentile and obesity as BMI >97^th^ percentile of the German reference system developed by Kromeyer-Hauschild et al.[35]. Cut-offs can be found in the online supplement (S1 Table).

#### Other covariates

Information on child and family sociodemographics, on birth and pregnancy, and on parental overweight and atopy were used to construct the propensity score and to adjust for in subsequent logistic regression analyses.

### Variables included in the propensity score

#### Sociodemographics

Municipality size (rural, small-town, urban, metropolitan), federal state of residence (16 states), migration background (none, one-sided, two-sided), country of birth (Germany/other country), year of birth (continuous), mother’s age at childbirth (continuous), presence of older siblings (yes/no), mother’s and father’s education (basic education, secondary education, high school degree), mother’s professional degree (no professional degree, vocational training, technical college, university of applied sciences, university).

#### Variables concerning birth and pregnancy

Twin birth (yes/no), maturity at birth (pre-term, term, post-term birth), birth weight (in quintiles), smoking of the mother during pregnancy (none, once in a while, regular), alcohol consumption of the mother during pregnancy (yes/no), gestational diabetes (yes/no), other complications during pregnancy (yes/no), breathing difficulties after birth (yes/no), infection after birth (yes/no), other complications after birth (yes/no), congenital malformation (yes/no), length of stay in hospital after birth (none, 1–7 days, 8–14 days, >14 days).

#### Variables concerning parental atopy

Allergic rhinitis (yes/no), atopic dermatitis (yes/no), and asthma (yes/no) of mother or father.

### Variables adjusted for in logistic regression analyses

The logistic regression analyses estimating the association between breastfeeding and overweight/obesity using the propensity score matched dataset were adjusted for age group (3–6 yrs, 7–10 yrs, 11–13 yrs, 14–17 yrs), sex (male/female) parental overweight (no parent, one parent, both parents) and socioeconomic status (low, middle, high). Socioeconomic status was measured using an index combining parents’ education and occupational qualification, occupational status and household net income which takes values between 3–21 points. It was estimated separately for both parents and study participants have been assigned either the higher of both point scores or in case of parents living apart the point score of the parent the child was living with primarily. A categorical variable distinguishing between three socioeconomic groups was built using quintiles of the distribution (low: 1. quintile; middle: 2.–4. quintile; high: 5. quintile), thus allowing to compare children and adolescents living in rather disadvantaged or in privileged circumstances with a broad group of study participants in the centre [36].

### Statistics

#### Use of propensity score methods to estimate the health effects of breastfeeding

In a randomized controlled trial, study participants in the treatment and control group should not differ substantially in their observed as well as in their unobserved baseline characteristics. However, in an observational study, some study participants are more likely than others to receive an intervention or to be exposed to a risk factor. For a thorough estimation of treatment effects, groups of treated and untreated subjects should be as similar as possible with regard to their baseline characteristics. A propensity score is defined as a conditional probability, which describes the likelihood of a person to receive an intervention given certain observed pretreatment characteristics [37–39]. Applications of the propensity score include matching, stratifying, or weighting on the score or using the score as a covariate in regression analyses [40,41].

We used propensity score matching and multivariable logistic regression analyses to estimate the effect of >4 months of breastfeeding compared with shorter or no breastfeeding on overweight and obesity. By matching on the propensity score, we sought to identify subsamples of breastfed and non-breastfed subjects with a comparable distribution of observed covariates. Among study participants matched on the propensity score, whether or not they have been breastfed is no longer dependent on measured baseline characteristics. We could thus achieve balance between the breastfed and the non-breastfed group and create a situation resembling that of a randomized trial, at least with respect to the observed covariates. The chosen approach helps to account for the heterogeneity between breastfed and formula-fed children when seeking to assess the effects of breastfeeding. Propensity scores can be estimated using logistic regression, with the intervention or exposure as the outcome variable and all the baseline characteristics that might have an impact on whether children have been breastfed as independent variables. Propensity scores describing the individual likelihood of having received an intervention or of being exposed to a risk factor with values between 0 (very unlikely) and 1 (very likely) can be generated for all study participants.

A key concept that propensity score methods rely on is that all covariates that influence whether a study participant has received an intervention or has been exposed to a risk factor have been considered and no unobserved confounding into the intervention or exposure remains [38]. Whether this assumption is met cannot be tested empirically, but has to be decided based on theoretical considerations [39]. It has been recommended to decide rather liberally in terms of including further variables when constructing a propensity score, because a rich set of covariates makes the assumption more credible. In addition, including irrelevant variables will not considerably harm the model, although it might slightly increase its variance. In contrast, omitting important variables will very likely result in biased results [38]. However, including variables that might have been affected by the intervention has to be carefully avoided. Matching on the propensity score offers several advantages over adjusting for observed background variables in regression analyses, among others the possibility of including a rich set of covariates without over-fitting the model and the possibility of checking covariate balance. However, matching and regression adjustment are not meant to be conflicting methods, but are best used in combination [38].

The propensity score for the probability that a participant has been breastfed for more than 4 months was estimated using logistic regression techniques. Study participants were matched using 1:1 nearest neighbor matching. This means one study participant in the breastfed group has been matched to one participant in the non-breastfed group who had approximately the same value on the propensity score. Matches have been built one at a time with the best available person, without striving to minimize the average absolute distance of all matched units on the propensity score (greedy matching) [39]. To avoid bad matches, it is possible to define a caliper, a maximum allowable difference between two matched participants. A caliper of 0.2 of the standard deviation of the logit of the propensity score has been recommended [38], we chose to impose an even closer matching range of 0.1. To estimate the impact of breastfeeding on overweight and obesity, we used multivariate logistic regression analyses and adjusted for age, sex, socioeconomic status, and parental overweight. Opinions differ on whether it is necessary to account for the matched nature of the dataset in the further estimation of the treatment effect by using statistical tests designed for matched data. We followed recommendations by Elizabeth Stuart [38], who argues one should pursue exactly the same analyses in the matched dataset one would choose when working with the unmatched data, because matching on the propensity score does not ensure that individual pairs will be well matched over all the included covariates. The analyses were conducted using SPSS 20 and PSMatching 3.03 [42]. To compare key characteristics of breastfed and non-breastfed study participants we used Student’s t test for continuous variables and the chi-square test for categorical variables. All p values are two-sided.

#### Propensity score model

A total of 28 covariates that might have influenced whether study participants had been breastfed were included in the model. Covariates were primarily chosen based on previous research findings on determinants of breastfeeding behavior [43,44]. In addition, we examined bivariate relations between potential covariates and found significant associations (*p*<0.05) in the majority of relations. Two sociodemographic variables (migration background and country of birth) and three variables concerning pregnancy and birth (gestational diabetes, other complications during pregnancy, and congenital malformation) were included based on theoretical considerations despite the fact that they did not show a significant association with breastfeeding behavior.

## Results

### Study participants

**Fig 1:**
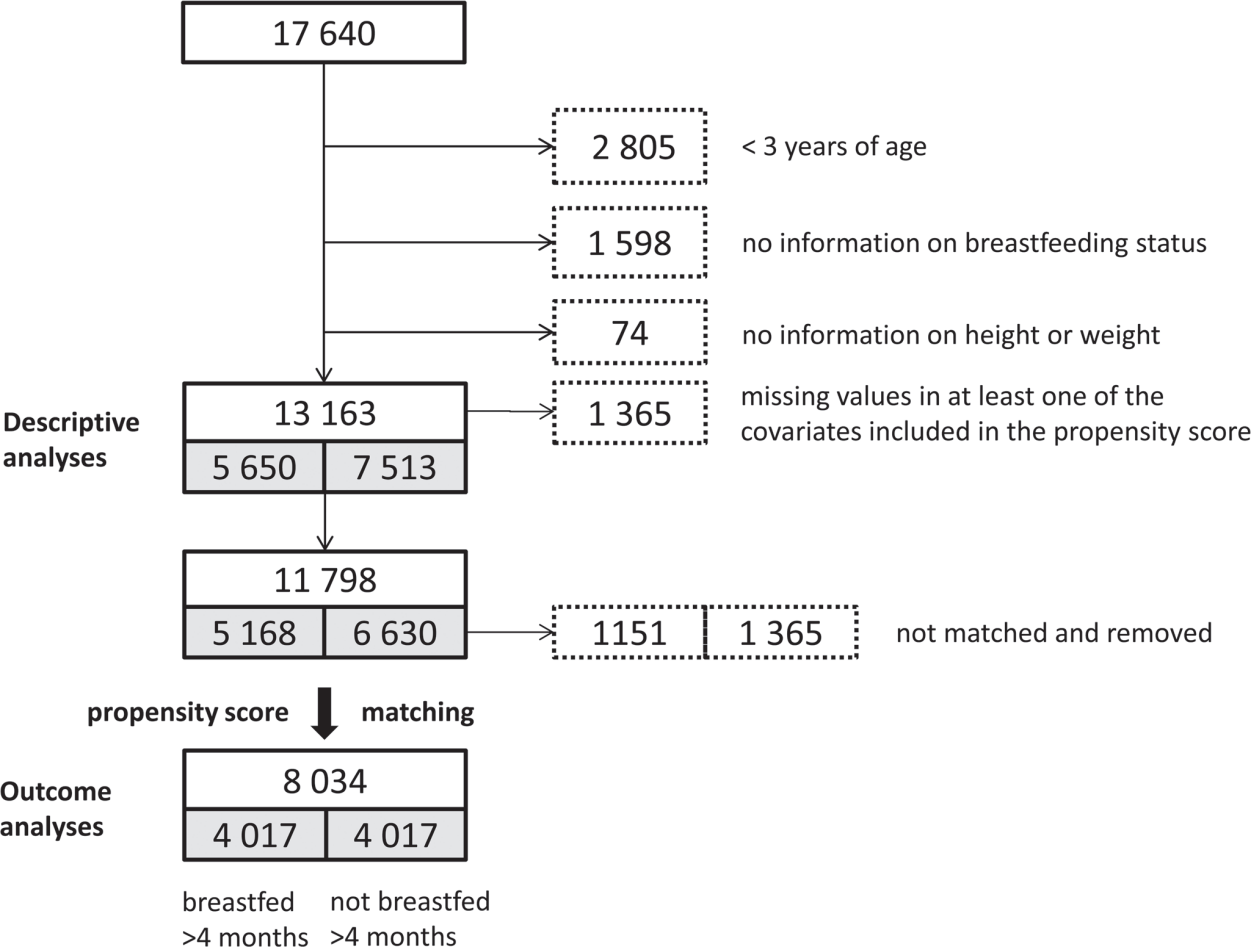
Flow chart of KiGGS study participants included in the analyses.

Of the 17640 KiGGS study participants we excluded 2805 participants who were under the age of 3 years, 1598 participants because of missing information on breastfeeding, and 74 participants because of missing information on height or weight (Fig. 1). This left 13163 study participants in the complete sample, of which 5650 participants (43%) had been breastfed for >4 months and 7513 (57%) had not. The study participants who were excluded due to missing data were significantly older, had a lower socioeconomic status and more often had a migrant background than those included in further analyses.

Prior to estimating the propensity score, we further excluded 1365 study participants who had missing values in any of the 28 covariates included in the score, because the software we used for propensity score matching does not accept datasets with missing values. Of the remaining 11798 study participants, 1151 of 5168 subjects in the exposed group (breastfed >4 months) and 2613 of 6630 subjects in the unexposed group (breastfed <4 months) could not be matched and were removed. After the matching procedure, 8034 participants (4017 matched pairs) remained in the dataset. The excluded participants were older and of lower socioeconomic status than the participants included in the matched dataset and more often had a migrant background.

### Descriptive data

Descriptive results for the breastfed and the control sample (n = 13163) can be found in Table 1. It is apparent from these findings that there are substantial differences in key sociodemographic features as well as in the prevalence of overweight and obesity between the two samples. Children who had been breastfed >4 months had a lower mean age than children with a shorter breastfeeding duration. Study participants with a longer breastfeeding duration had a high socioeconomic status considerably more often than those with shorter breastfeeding times. Prevalence of overweight and obesity was significantly lower in subjects with a longer breastfeeding duration.

**Table 1:** Characteristics of the 13163 study participants, according to breastfeeding status.

**Variable**	**Breastfed >4 months**		**p value** ^a^
	*yes (n = 5650)*	*no (n = 7513)*	
Age in years (mean (SD))	9.06 (4.10)	10.35 (4.19)	<0.001
Mother’s age at childbirth(mean (SD))	29.43 (4.69)	27.87 (5.14)	<0.001
Female sex, n (%)	2840 (48.3)	3628 (50.3)	0.025
Socioeconomic status, n (%)			<0.001
low	511 (9.1)	1345 (18.1)	
medium	3160 (56.3)	4778 (64.1)	
high	1945 (34.6)	1326 (17.8)	
Migration background, n (%)			0.464
none	4582 (81.4)	6067 (80.9)	
one-sided	393 (7.0)	507 (6.8)	
two-sided	655 (11.6)	923 (12.3)	
Older siblings ≥1, n (%)	3077 (54.5)	3867 (51.5)	0.001
Smoking of mother during pregnancy, n (%)			<0.001
regularly	103 (1.8)	473 (6.4)	
once in a while	407 (7.3)	1223 (16.5)	
Parental overweight, n (%)			<0.001
both parents	1049 (18.7)	1754 (23.6)	
one parent	2419 (43.1)	3347 (45.0)	
Overweight, n (%)	658 (11.6)	1304 (17.4)	<0.001
Obesity, n (%)	235 (4.2)	555 (7.4)	<0.001

^a^To obtain p values we used t-tests for continuous variables (age in years, mother’s age at childbirth) and chi-square test for categorical variables; p values are two-sided.

### Assessing the quality of the matched sample

**Fig 2:**
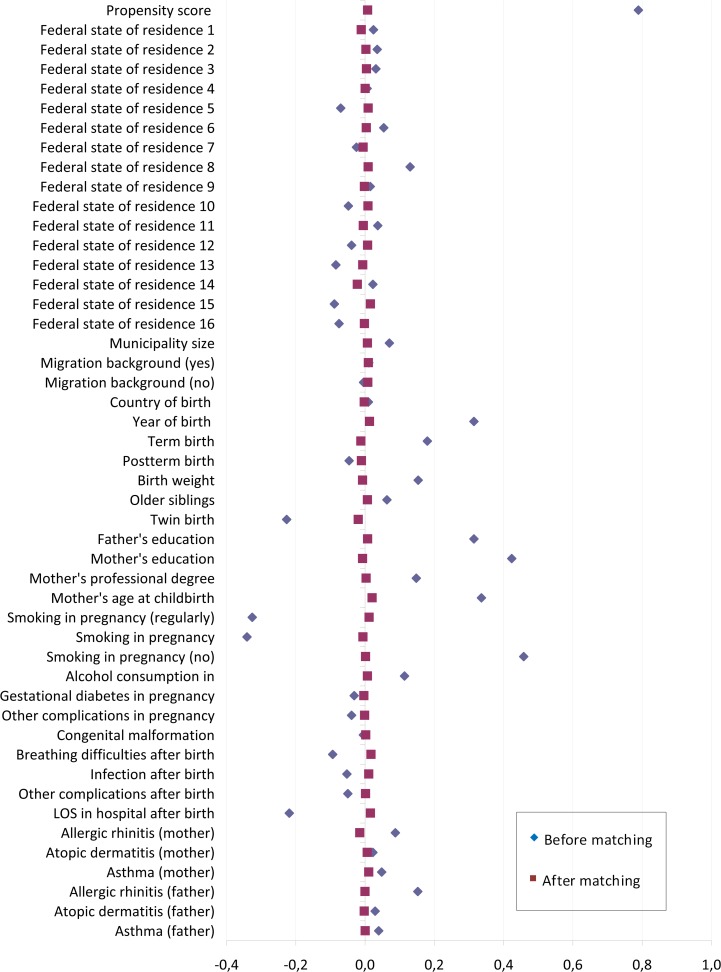
Standardized differences before and after matching. Dotplot of standardized mean differences (Cohen’s d) for the covariates included in the propensity score before and after matching.

**Fig 3:**
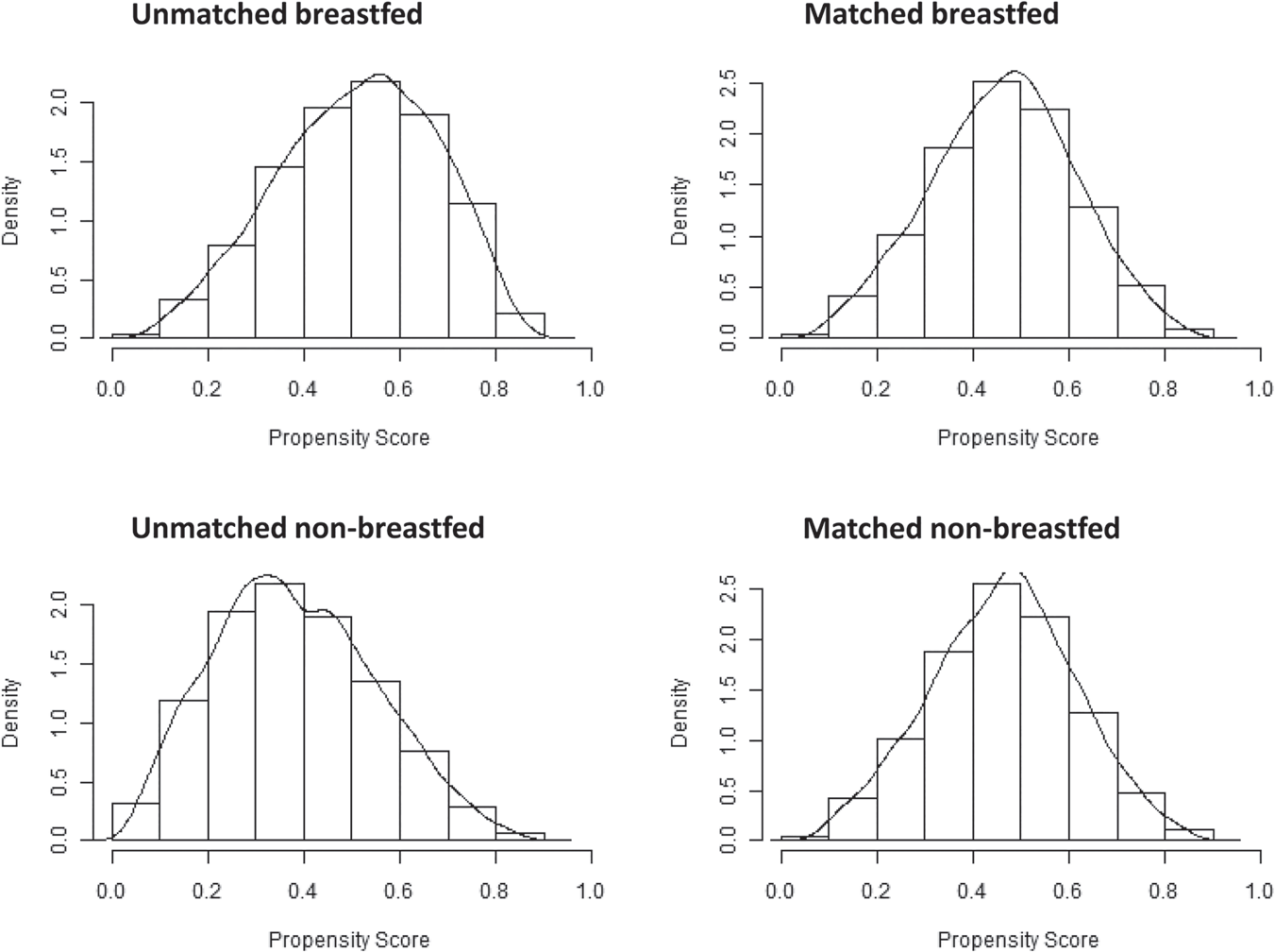
Distribution of propensity scores before and after matching. Distribution of propensity scores with overlaid kernel density estimate of study participants in the breastfed and non-breastfed groups before and after the matching procedure.

**Fig 4:**
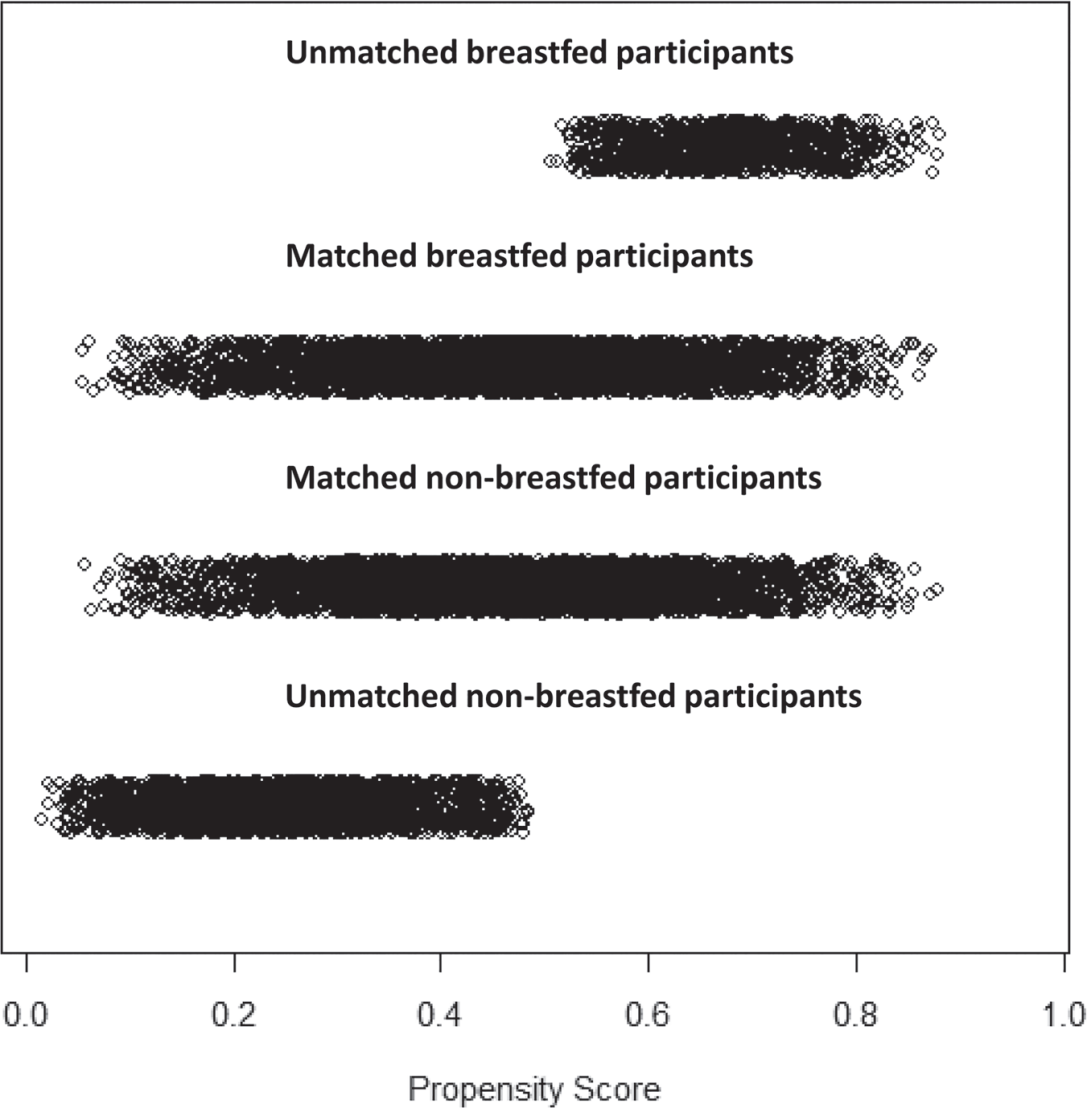
Distribution of propensity scores in matched and unmatched groups. Distribution of propensity scores of study participants in the breastfed and non-breastfed groups who could be matched and remained in the dataset and of those who could not be matched and were removed.

After matching, we carefully assessed the adequacy of the model and the matched sample according to prior recommendations [38,39]. The overall Chi square balance test did not indicate statistical significance (Chi^2^ = 11.05, p = 1.00). Unfortunately, the L1 imbalance measure was not smaller in the matched sample than in the unmatched sample (1.00 vs. 1.00), thus not indicating better overall balance. Standardized mean differences (Cohen’s d) of all the included covariates were reduced after matching, the largest remaining difference being a value of d = 0.04 for length of stay in hospital after birth (Fig. 2). The distribution of the propensity scores in the unmatched and matched breastfed and non-breastfed groups also showed that covariate balance improved through the matching procedure (Fig. 3). There was sufficient overlap between the breastfed and the non-breastfed group and the region of common support ranged over almost the entire distribution of the propensity score. Only participants on the upper and lower margins of the distribution remained unmatched and were removed from the dataset (Fig. 4).

### Association between breastfeeding and overweight/obesity

Table 2 presents odds ratios (ORs) of the association between breastfeeding and overweight or obesity at age 3–17 years. Bivariate analyses in the unmatched data suggest a substantial reduction in the risk of both overweight and obesity for infants who were breastfed (OR 0.63 [95% CI 0.57–0.69] for overweight and OR 0.54 [95% CI 0.47–0.64] for obesity). Analyses of the matched dataset adjusted for age group, sex, socioeconomic status, and parental overweight still indicated a relevant association between breastfeeding and overweight and obesity, but the protective effect was considerably smaller than in the unadjusted analyses (OR 0.81 [95% CI 0.71–0.92] for overweight and OR 0.75 [95% CI 0.61–0.92] for obesity).

**Table 2:** Association between breastfeeding and childhood overweight and obesity.

	Unmatched sample,unadjusted analyses(n = 13163)		Matched sample, unadjusted analyses*n = 8034		Matched sample, adjusted analyses*n = 8034		
**Overweight**							
	*OR (95% CI)*	*p-value*	*OR (95% CI)*	*p-value*	*OR (95% CI)*		*p-value*
Breastfeeding		<0.001		<0.001			0.002
<4 months	1		1		1		
≥4 months	0.63 (0.57–0.69)		0.76 (0.67–0.86)		0.81 (0.71–0.92)		
Age							<0.001
3–6 years					1		
7–10 years					1.96 (1.63–2.36)		
11–13 years					2.24 (1.84–2.74)		
14–17 years					2. 09 (1.71–2.55)		
Sex							0.541
male					1		
female					0.96 (0.84–1.09)		
Socioeconomic status							<0.001
low					1		
middle					0.73 (0.61–0.89)		
high					0.53 (0.42–0.66)		
Parental overweight							<0.001
both parents					1		
one parent					0.47 (0.40–0.54)		
no parent					0.23 (0.20–0.28)		
**Obesity**							
	*OR (95% CI)*	*p-value*			*OR (95% CI)*	*p-value*	
Breastfeeding		<0.001		<0.001		0.005	
<4 months	1		1		1		
≥4 months	0.54 (0.47–0.64)		0.69 (0.57–0.85)		0.75 (0.61–0.92)		
Age						<0.001	
3–6 years					1		
7–10 years					2.01 (1.49–2.72)		
11–13 years					2.04 (1.47–2.81)		
14–17 years					2.86 (2.10–3.88)		
Sex						0.540	
male					1		
female					0.94 (0.77–1.15)		
Socioeconomic status						<0.001	
low					1		
middle					0.69 (0.52–0.90)		
high					0.45 (0.32–0.63)		
Parental overweight						<0.001	
both parents					1		
one parent					0.40 (0.32–0.49)		
no parent					0.19 (0.14–0.26)		

*adjusted for age group, sex, socioeconomic status, and parental overweight

Because we included a broad age range of 3- to 17-year-old children and adolescents, we further examined whether the effect of breastfeeding on overweight and obesity differed between age groups. Logistic regression analyses stratified by age groups indicated that the protective effect of breastfeeding is largest at the age of 7–10 years (Table 3). No significant effect of breastfeeding on overweight or obesity could be seen in children under the age of 7 years or in adolescents aged 11–17 years. In children aged 3–6 years we saw a non-significantly decreased risk for breastfed children to be obese, but the number of subjects in this subgroup might to be too small to detect a significant effect.

**Table 3:** Association between breastfeeding and childhood overweight and obesity, stratified by age groups.

		****Overweight****			****Obesity****		
Age group	*Breastfeeding*	*n*_*overweight*_ *(n*_*complete*_ *)*	*OR (95% CI)*	*p-value*	*n*_*obese*_ *(n*_*complete*_ *)*	*OR (95% CI)*	*p-value*
3–6 years				0.973			*0*.*743*
	<4 months	102 (1123)	1		40 (1123)	1	
	≥4 months	95 (1109)	1.01 (0.75–1.35)		28 (1109)	0.74 (0.45–1.22)	
7–10 years				0.001			0.002
	<4 months	222 (1196)	1		90 (1196)	1	
	≥4 months	153 (1249)	0.67 (0.53–0.84)		50 (1249)	0.56 (0.39–0.81)	
11–13 years				0.184			0.965
	<4 months	151 (803)	1		49 (803)	1	
	≥4 months	127 (826)	0.84 (0.64–1.09)		45 (826)	0.99 (0.64–1.52)	
14–17 years				0.166			0.255
	<4 months	152 (895)	1		74 (895)	1	
	≥4 months	120 (833)	0.83 (0.63–1.08)		56 (833)	0.81 (0.56–1.17)	

*matched sample (n = 8034), adjusted for sex, socioeconomic status, and parental overweight

## Discussion

Results from our propensity score-matched analyses suggest that full breastfeeding for the infant’s first 4 months of life might help to prevent overweight and obesity in later childhood. We found a considerable reduction in the risk of overweight and an even greater reduction in the risk of obesity for children who had been breastfed for more than 4 months compared with those who had not been breastfed or who had been breastfed for less than 4 months. These findings indicate a stronger association of breastfeeding and overweight than has been reported in two previous studies that applied propensity score methods to estimate the protective effect of breastfeeding on childhood obesity and found only weak or insignificant associations [30,31]. Studies from countries where breastfeeding and obesity are less socially patterned and studies using sibling comparisons likewise found rather small or no significant effects [23–29]. The authors of a recent meta-analysis concluded that breastfeeding leads to a reduction in the prevalence of later overweight and obesity of about 10%, if only studies with large sample sizes and sufficient control for confounding were considered [6], which is a somewhat smaller effect than we found in our data. Age-adjusted analyses of our data showed that the protective effect of breastfeeding was visible only in children aged 7–10 years, while in children under the age of 7 years and in adolescents between 11 and 17 years no statistically significant association between breastfeeding and overweight or obesity could be demonstrated. This is largely consistent with the results of a recent meta-analysis initiated by the World Health Organization, which found the strongest effects of breastfeeding in children and adolescents aged 10–19 years, smaller effects in children aged 1–10 years, and only a slight impact in subjects over the age of 20 years [6] although the peak impact of breastfeeding in our sample was seen in slightly younger children. Prior analyses of KiGGS data showed that the prevalence of overweight in children seems to rapidly increase between the age of 5 and 8 years. The authors presume that this might be attributed to the transition between kindergarten and school entry [45]. The sudden increase in the prevalence of overweight after school entry seems to take place independently of children’s socioeconomic status [45]. Considering the comparatively strong association between breastfeeding and overweight we found in this age group it may be that early childhood factors appear to have the strongest impact in a group that is especially vulnerable to become obese anyway. Even though public health interventions for children in primary school age that are based on regular physical activity and healthy food choices might be the most promising preventive measures, our findings suggest that encouraging and enabling mothers to breastfeed their children might also help to tackle childhood obesity. In Germany, currently around half of all mothers choose to fully breastfeed their infants for at least 4 months [44] as has been recommended by the World Health Organization [46]. However, a mother’s decision to breastfeed is based on multiple different personal and social factors and choosing to breastfeed often requires her to accept considerable constraints with regard to work and employment as well as to other activities in the months following childbirth [25]. Health care professionals should thus try to carefully incorporate mothers’ priorities and wishes into counseling on infant nutrition. As in many other affluent countries there is a strong social gradient in both breastfeeding initiation and duration [44]. Further research activities should also focus on why this is so and subsequently develop targeted interventions to reduce the socioeconomic differences in breastfeeding.

### Strengths and limitations

Our study has four key strengths. We used data from a nationally representative sample of children and adolescents living in Germany, which makes our findings widely transferable. By matching on the propensity score we were able to minimize confounding of breastfeeding behavior to the greatest possible extent and to create a situation similar to that of a randomized controlled trial, at least with regard to the measured covariates included in the score. We included a broad range of covariates that might have influenced whether and how long infants were breastfed and we adjusted for a multidimensional measure of family socioeconomic status, which merged parental education, occupational qualification, occupational status, and net income into a single index.

Despite these strengths, our findings have to be interpreted in the context of certain limitations. Concerning possible recall bias of breastfeeding, which might be expected owing to the retrospective data acquisition that covered recall periods very much differing in length, prior studies showed that mothers recalled quite accurately for how long they had breastfed their children even in the long term [47–49]. Women who breastfed for a shorter period tended to overestimate and women who breastfed for a longer period tended to underestimate breastfeeding duration, thus any potential recall bias would be more likely to attenuate estimates of breastfeeding benefits [48,49]. Even though we made considerable efforts to control for confounding, we could only consider baseline covariates collected in the survey. Unobserved confounding remains a tremendous statistical challenge in handling observational data and might have led to overestimations of breastfeeding benefits in our analyses. We did for example miss information on mothers’ occupational situation before and after childbirth and on social support mothers and parents experienced during this time, which might highly influence decisions on breastfeeding initiation and duration. Furthermore, information on the factors that might have influenced whether and how long infants were breastfed and on breastfeeding behavior itself was collected retrospectively. We aimed to include only those covariates into the propensity score that are sufficiently stable over time to presume that they had been valid already at the time of childbirth. Covariates not allowing this assumption, for example mothers’ weight, were excluded in the construction of the propensity score. Additional analyses of the study participants who were excluded because of missing values and those who could not be matched and were removed after the matching procedure showed that they were older, of lower socioeconomic status and more often had a migration background than the children and adolescents included in the analyses, which is not unexpected but might further restrict the interpretation of our findings and their generalizability on the population level. Because of the constraints of the software used, we dichotomized the exposure into more or less than 4 months of breastfeeding, which might have further diminished estimates of the true effects of breastfeeding. In addition, we were thus unable to draw any conclusions on a possible dose-response relationship, differentiating between initial breastfeeding and 4 or 6 months of breastfeeding.

## Conclusion

Our findings support the hypothesis that breastfeeding has a protective effect on childhood overweight and obesity, which is visible especially in children of primary school age. The results suggest that the promotion of breastfeeding might help to reduce the prevalence of obesity among children, even though it may be of minor importance compared to public health interventions related to physical activity, healthy food choices and the underlying social, economic and cultural factors. Like the majority of previous studies, which reported heterogeneous results, our analyses rely on observational data. Even though we tried to minimize potential confounding by using propensity score methods and by considering a rich set of covariates, we have to remain cautious when interpreting the evidence on the beneficial effects of breastfeeding to inform public health interventions and guidelines on infant nutrition.

## Supporting Information

S1 Table Cut-offs for overweight and obesity for boys and girls aged 3–17 years. Overweight is defined as BMI>90^th^ percentile, obesity as BMI>97^th^ percentile. The reference system is based on pooled data from 17 surveys conducted in Germany between 1985 and 1999 (K. Kromeyer-Hauschild, M. Wabitsch, D. Kunze et al.: Monatsschr. Kinderheilk. 149 (2001) 807–818.).
(XLSX)
