# Correction: Does Breastfeeding Help to Reduce the Risk of Childhood Overweight and Obesity? A Propensity Score Analysis of Data from the KiGGS Study

**DOI:** 10.1371/journal.pone.0126675

**Published:** 2015-04-16

**Authors:** Maike Miriam Grube, Elena von der Lippe, Martin Schlaud, Anna-Kristin Brettschneider

There is an error in the first sentence of the Results section in the Abstract. The correct sentence is: Adjusted analyses of the matched dataset (n = 8034) indicated that children who were breastfed for >4 months had a significant reduction in the odds of overweight (OR 0.81 [95% CI 0.71–0.92]) and obesity (OR 0.75 [95% CI 0.61–0.92]) compared to children who were not breastfed or who were breastfed for a shorter duration.

## References

[1] Grube MM, von der Lippe E, Schlaud M, Brettschneider A-K (2015) Does Breastfeeding Help to Reduce the Risk of Childhood Overweight and Obesity? A Propensity Score Analysis of Data from the KiGGS Study. PLoS ONE 10(3): e0122534 doi: 10.1371/journal.pone.0122534 25811831 10.1371/journal.pone.0122534 PMC4374721

